# Biocompatible Photocrosslinked Chitosan- and Gelatin-Based Hydrogels for Wound Healing Applications

**DOI:** 10.3390/gels12040290

**Published:** 2026-03-29

**Authors:** Isabella Nacu, Andreea Vasilache, Catalina Anisoara Peptu, Liliana Verestiuc, Andreea Luca

**Affiliations:** 1 Department of Biomedical Sciences, Faculty of Medical Bioengineering, “Grigore T. Popa” University of Medicine and Pharmacy, 700115 Iasi, Romania; isabella.nacu@umfiasi.ro (I.N.); andreea.loredana069@gmail.com (A.V.); andreea.luca@umfiasi.ro (A.L.); 2 Cristofor Simionescu Faculty of Chemical Engineering and Environmental Protection, Gheorghe Asachi Technical University of Iaşi, 700050 Iasi, Romania; catipeptu@yahoo.co.uk

**Keywords:** hydrogels, methacrylated chitosan, methacrylated gelatin, allylic gelatin, wound healing, drug delivery

## Abstract

The study presents novel photocrosslinked hydrogels based on methacrylated chitosan and methacrylated gelatin/allyl-modified gelatin and compares their properties as drug delivery systems in wound healing applications. The polymers were selected due to their biocompatible, mucoadhesive, cell-interactive properties and flexibility in adjusting their structure, making them suitable candidates for applications that require tissue repair. A range of hydrogel formulations was obtained by modulating the ratio of modified chitosan to two distinct modified gelatins, with photocrosslinking performed using Irgacure 2959 as the photoinitiator. FT-IR analysis, SEM data, and swelling and mechanical properties confirmed the 3D networking and the compatibility between the hydrogel components. Allylic gelatin-based hydrogels present larger pores and a stronger pH-responsive swelling behaviour compared to methacrylated gelatin-based samples, reflecting the higher flexibility of allylic gelatin networks. The hydrogels release bacitracin during the first six hours, with a release profile that follows a non-Fickian diffusion mechanism. Cytocompatibility and wound healing potential were tested in the presence of human and mouse fibroblasts, cells with a pivotal role in the wound healing process. All formulated hydrogels exhibit antioxidant capacity and protein stabilization properties, which are attributed to the presence of chitosan in their composition. The cytocompatibility, in vitro wound healing, and biological properties of the obtained hydrogels, as well as the drug release results, confirm their suitability in wound healing applications.

## 1. Introduction

Wounds are disruptions in skin integrity induced by physical trauma (cuts, scrapes, burns, punctures) or underlying chronic conditions. Accidents, surgeries, and environmental injuries cause acute wounds, while chronic wounds are often associated with diabetes, pressure ulcers, poor circulation, infection, or severe inflammation. The natural barriers of the skin are compromised in a wound, and the blood or the clots formed during the initial phases of hemostasis serve as a nutrient-rich medium for the rapid proliferation of invasive bacteria that infiltrate the wound, exacerbating pain, hindering the healing process, and potentially leading to severe systemic symptoms [1]. The increased incidence, prevalence, morbidity, and mortality rates of skin diseases have positioned them at the frontline of global public health challenges, and new intervention strategies, emergent technologies, and innovative wound dressing products have been considered in mitigating the impact of skin wounds [2].

Wound repair is a complex and dynamic biological process that is highly regulated and involves the interaction of various biological pathways. Restoring tissue integrity is based on four interconnected phases: hemostasis, inflammation, proliferation, and remodelling [3]. This multi-layered process involves coordinated cellular activity (platelets, neutrophils, macrophages, fibroblasts) and molecular signalling to replace damaged tissue, often resulting in scar formation [4].

Skin tissue engineering and regenerative medicine have significantly advanced the treatment of wounds by developing artificial or bio-artificial constructs that mimic the structures and biological functions of natural tissues and are able to stimulate repair processes [5]. Moreover, antimicrobial dressings have been prepared with the inclusion of drugs with antimicrobial/antifungal properties as a strategy to mitigate wound infections [6]. In this context, polymeric hydrogels have demonstrated a very good capacity to reproduce the extracellular matrix (ECM), to protect the wound against microbial infection, reduce fluid loss, promote oxygen delivery to the lesion, and overall improve the healing process [7,8]. In addition, the porous structure of hydrogels allows for an efficient exchange of nutrients and wastes and has a positive effect on the adhesion, proliferation, migration, and differentiation of various cells such as keratinocytes, fibroblasts, and mesenchymal stem cells [9]. Based on the origin of the polymer used, hydrogels with natural or synthetic characteristics have been developed, and a large number of polymers have been tested in order to obtain materials that absorb exudates and stimulate the remodelling of the tissue in the wound bed [10,11]. Natural hydrogels are extensively tested for their low toxicity, biocompatibility, and biodegradability, and various polymers have demonstrated their performance in wound dressing applications: collagen [12], gelatin [13], cellulose [14], chitosan [15,16], hyaluronic acid [17], sodium alginate [18], xanthan [19], and gellan [20].

Chitosan is a biodegradable and non-toxic polysaccharide, a deacetylated form of chitin that includes two monomers (2-acetamido-2-deoxy-D-glucopyranose and 2-amino-2- deoxy-D-glucopyranose units) and contains at least 50% free amine. It has excellent biocompatibility, mucoadhesive properties, and non-immunological reactions. The polymer’s antimicrobial qualities, ability to stimulate blood coagulation, collagen deposition, and fibroblast proliferation are valuable properties in wound healing applications. In addition, chitosan is a versatile biopolymer, and its biological properties can easily be adjusted by modifying its structure or functionality [21]. Considering these advantages, chitosan is considered a promising product for burn and wound care treatment, in combination with proteins or bioactive molecules [22]. Chitosan functional groups (amino groups –NH_2_ and hydroxyl groups –OH) allow for extensive chemical modifications, such as carboxymethylation, quaternization, and methacrylation, to enhance solubility and functionality [23]. RGD peptide functionalization or collagen blending has been employed to provide a conducive environment for cell adhesion in chitosan-based hydrogels [24].

On the other hand, gelatin, a hydrolysis product of collagen, the major structural protein of connective tissues, is a flexible protein, biocompatible with human tissues, which can be modified/functionalized with reactive groups. The molecular weight and properties of gelatin are strongly dependent on collagen origin and the manufacturing process (enzyme denaturation method, or extraction in acidic (gelatin type A) or alkaline (gelatin type B) conditions) [25]. The repeating amino acid sequence (Gly–X–Y) and the comparatively high concentration of hydroxyproline and hydroxylysine typical for collagen are preserved in gelatins. Gelatin presents various reactive groups (–COOH and –NH_2_) on the side chains and good water solubility, facilitating protein modification. Several modified gelatin types were methacrylated, such as GelMA [26], and used to obtain scaffolds for skin, bone, and articular cartilage matrix [27]. Gelatin modified with methacrylate groups (GelMA) leads to hydrogels for biomedical purposes due to the crosslinking possibilities over a wide range of light wavelengths [28]. In the last decade, GelMA was extensively tested in tissue engineering owing to its superior biocompatibility and capacity to enhance cell adhesion [29]. Recently, another gelatin derivative, allyl-modified gelatin, was considered as a 3D supportive hydrogel for long-term culture of fibroblasts in regenerative medicine [30]. Moreover, allyl-modified gelatin has been tested as a hydrogel precursor when reacting with thiol crosslinkers, being used especially in 3D bioprinting applications [31,32].

The most frequently used initiators in photopolymerization reactions are free radical photoinitiators, type I and II, and 2-hydroxy-4-(2-hydroxyethoxy)-2-methylpropio-phenone (Irgacure 2959) is the common type I photoinitiator [33]. The side groups on the gelatin chains are polymerized using UV light, and produce covalently crosslinked networks with improved mechanical stiffness and stability [34,35]. The association between hydrogels and different pharmacological compounds is expected to protect and help the wound healing process.

Considering the described aspects, the aim of this study was to develop novel photocrosslinked hydrogels based on methacrylated chitosan and methacrylated gelatin/allyl-modified gelatin, and to compare their suitability as drug delivery systems in wound healing applications. The novelty of this work consists of combining allyl-modified gelatin and methacrylated chitosan as an alternative to methacrylated gelatin—based hydrogels. To our knowledge, no combination based on allyl-modified gelatin and methacrylated chitosan was reported in the literature as hydrogels for tissue engineering.

## 2. Results and Discussion

### 2.1. FT-IR Spectroscopy

**Figure 1:**
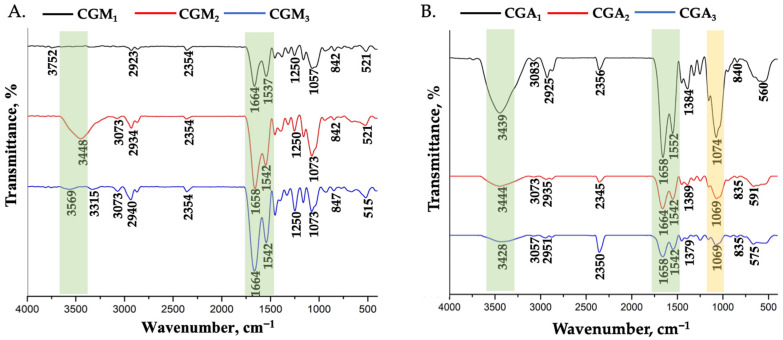
FT-IR spectra of the hydrogels: (**A**) CGM_1_, CGM_2_, CGM_3_; (**B**) CGA_1_, CGA_2,_ CGA_3_.

Hydrogels based on methacrylated chitosan and methacrylated gelatin/allyl-modified gelatin have been prepared by mixing polymers with a photoinitiator and exposure to UV light (λ = 365 nm). Figure 1 presents the FT-IR spectra of the CGM (CGM_1_–CGM_3_) and CGA (CGA_1_–CGA_3_) samples recorded in the 4000–500 cm^−1^ range. All spectra exhibit a centred absorption band at ~3430–3560 cm^−1^, associated with O–H and N–H stretching vibrations, representative for hydrogen bonding within the polymeric network. These vibrations are more intense in CGM_1_ and CGA_1_, consistent with their higher gelatin content compared to chitosan.

The absorption bands at 2935–2940 cm^−1^ are attributed to C–H stretching vibrations from the aliphatic –CH_2_ and –CH_3_ groups, present on both gelatin and chitosan backbones [36]. Pronounced peaks in the 1670–1550 cm^−1^ region are assigned to amide I (~1658 cm^−1^, C=O stretching) and amide II (~1540–1552 cm^−1^, N–H bending coupled with C–N stretching) vibrations, confirming the presence of gelatin in all formulations. The higher intensity of these amide bands in CGM_1_ and CGA_1_ further supports the increased gelatin concentration in the final hydrogels [37,38].

Importantly, in the CGM scaffolds, an increased absorption around 1650 cm^−1^ can be attributed to the stretching vibration of C=C bonds from some unreacted methacrylate groups grafted onto chitosan, partially overlapping with the amide I band. Additionally, characteristic bands in the ~840 cm^−1^ region are associated with bending vibrations of =C–H groups and confirm the presence of methacrylate functionalities.

Similarly, the CGA spectra exhibit weak but distinguishable contributions in the 1658 cm^−1^ and ~1069 cm^−1^ regions, which are assigned to C=C stretching and =C–H bending vibrations of allyl groups added onto the gelatin backbone. These observations confirm the successful chemical functionalization of gelatin and chitosan prior to hydrogel formation [39].

Overall, the FT-IR results demonstrate the existence of modified gelatin and chitosan within the hydrogel networks, while the presence of allyl or methacrylate-specific bands confirms successful functionalization and explains the compositional and structural differences observed among the hydrogels.

### 2.2. Hydrogels Morphology

The morphology and porosity of the hydrogels are critical factors that impact cell adhesion and proliferation. The crosslinking of the polymer chains is an essential aspect in 3D networks’ stability, also impacting their ability to respond and interact in the specific tissue environment [40]. Allyl-modified gelatin inclusion in hydrogel composition is expected to modify the crosslinking reaction and density of the network, as this polymer can offer mild reinforcement for the hydrogels’ structure due to the lower reactivity of the allyl group [41]. Methacrylated chitosan associated with allylic gelatin combines different polymerization behaviour of the two double bonds, resulting in lower crosslinking density, as compared to methacrylated chitosan–methacrylated gelatin, where both polymers present high reactivity group [42]. The presence of allylic gelatin in the resulting hydrogels contributes to a better control of the polymerization process, a flexible network, while still providing gelatin-specific cell adhesion motifs, assuring an environment appropriate for cell interaction.

The pore dimensions are directly implicated in nutrient and oxygen diffusion, waste elimination, and cell migration, by forming a suitable microenvironment and stimulating cell–cell communication, pivotal in wound healing processes. Smaller pores, with dimensions ranging from 80 to 120 µm, present a beneficial effect in the inflammatory stages of wound healing, while larger pore dimensions (200 µm) will have a positive impact in the revascularization stages [43]. In this study, the morphology of hydrogels was analyzed using SEM. The hydrogels based on modified gelatin presented wider pores (up to 200 µm) and, as a consequence, a more fragile structure, compared with the modified chitosan-based hydrogel, which presented smaller pores (up to 160 µm). The study of Bucciarelli et al. showed that the pore size increases with the concentration of methacrylated chitosan, due to the presence of a higher number of fast-responsive active groups in the crosslinking process, stabilizing the pore dimensions [44].

**Figure 2:**
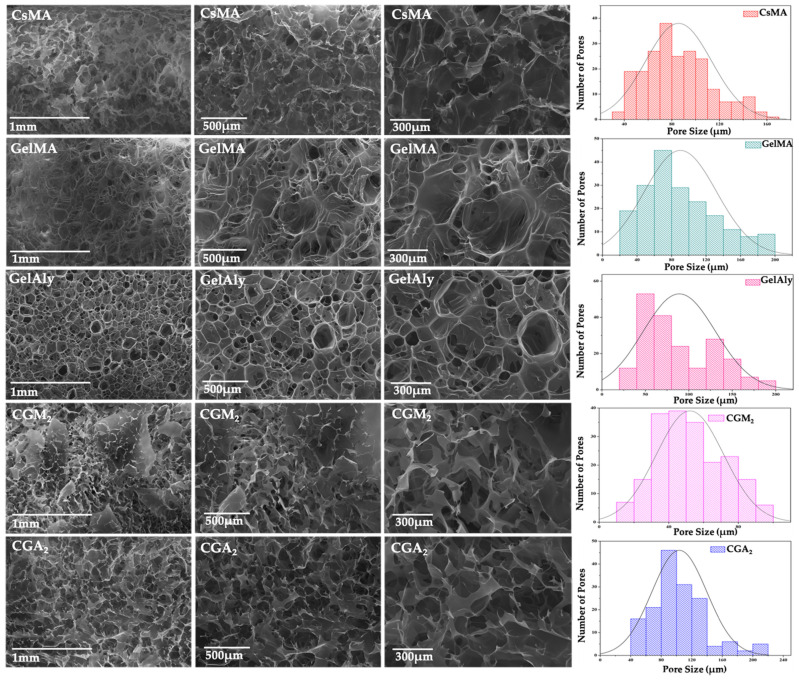
Images of SEM and pore size distribution for CsMA, GelMA, GelAly, CGA_2_, and CGM_2_ hydrogels.

The chemical cross-linking, via methacrylated groups, remodelled the pore distribution, and the pore size was reduced in hybrid hydrogels. As can be observed in Figure 2, the inclusion of methacrylated chitosan downsized the porosity of 3D hydrogels due to an increased number of interactions between the methacrylic groups.

The pore size values in hydrogels with modified chitosan and GelMA ranged from 15 to 95 µm, while for hydrogels based on GelAly ranged from 45 to 210 µm (Table 1). The higher pore dimension was registered for the CGA_2_ hydrogels, being an effect of a less dense network structure of the allylic gelatin. The degree of GelMA substitution and the position of the active groups on the polymeric chain influenced the photo-crosslinking and contributed to the differences in pore dimensions.

Both photocrosslinking and addition of methacrylic groups from the CsMA structure contributed to the formation of covalent bonds and produced interconnected, uniformly distributed pores, which favor the diffusion of nutrients [36]; for the CGM_2_ hydrogels, the majority of pores are within the ranges considered optimal for supporting skin regeneration, which are 20–120 µm [45].

### 2.3. Swelling Behaviour in Simulated Physiological Conditions

**Figure 3:**
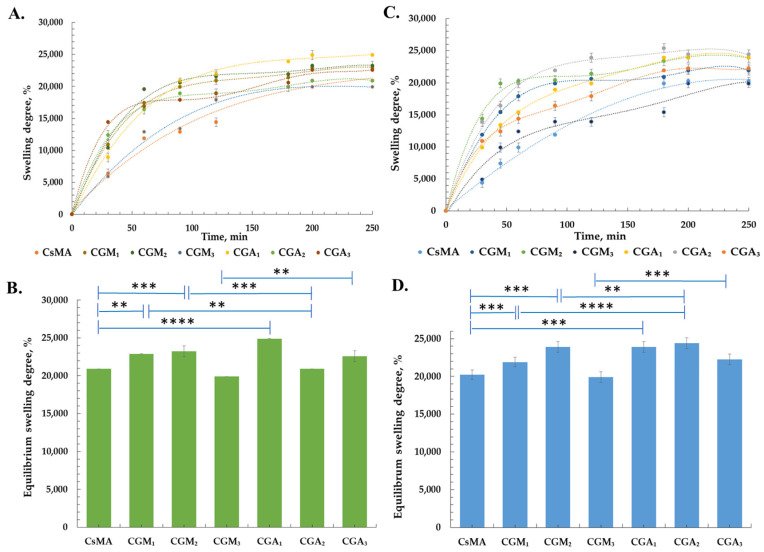
Swelling behaviour and equilibrium swelling degree of the CGM and CGA hydrogels, at pH 5.4 (**A**,**B**) and pH 7.2 (**C**,**D**). Values were expressed as the mean ± SD (*n* = 3). ** *p* < 0.01, *** *p* < 0.001, **** *p* < 0.0001 (ANOVA, two-ways).

Figure 3 illustrates the time-dependent swelling behaviour and the equilibrium swelling degree of CsMA, CGM, and CGA hydrogels after immersion in phosphate buffer solutions (PBS, pH 7.2 or pH 5.4, 0.01 M). To assess water uptake ability, the hydrogels were incubated in two different types of PBS (pH 5.4 and pH 7.2) at 37 °C for 2 h and allowed to reach the state of equilibrium. The 5.4 and 7.2 pH values of PBS solutions were chosen because the natural pH of the healthy human epidermal layer is slightly acidic, with pH values from 4.5–5.3 to 6.8, when reaching the lower stratum corneum, while wounds have a slightly alkaline microenvironment due to trauma, exposing underlying tissues with a pH of 7.2–7.44 [46,47]. However, alkaline microenvironments are beneficial for bacterial growth [48].

The macroporous morphology and the presence of various hydrophilic functional groups (–COONa, –OH, –NH_2_, and –CONH–) in the 3D networks are determinant features for the swelling properties [49]. At pH 5.4, the hydrogels exhibit comparatively lower swelling degrees, which can be attributed to the partial protonation of functional groups and the resulting reduction in electrostatic repulsion within the polymer network [50].

Within the CGA series, CGA_1_ and CGA_2_ show higher swelling values than CGA_3_, consistent with their higher gelatin content, with increased possibilities for crosslinking. A similar trend was observed for the CGM series, although overall swelling remains more restricted due to the presence of methacrylated chitosan [51].

At pH 7.2, all samples demonstrated an increased swelling rate and equilibrium swelling degree, associated with enhanced ionization of functional groups, leading to network expansion. CGA hydrogels exhibit a stronger pH-responsive swelling behaviour compared to CGM samples, reflecting a higher flexibility of allylic gelatin networks. In contrast, CGM hydrogels showed a more controlled swelling response, which can be attributed to the crosslinking of methacrylate functionalities on chitosan [51]. The CsMA hydrogel displays intermediate swelling behaviour at both pH values, serving as a benchmark for evaluating the influence of the gelatin content.

The equilibrium swelling behaviour of the hydrogels showed a strong correlation with their pore size characteristics. Samples exhibiting larger average pore sizes and broader pore size distributions generally display higher equilibrium swelling degrees, as the increased pore volume facilitates water penetration and retention within the hydrogel network [49,52]. In particular, CGA hydrogels demonstrated higher equilibrium swelling compared to CGM samples. This behaviour can be explained by an open network associated with allylic gelatin mobility, which enhanced water uptake. CGM hydrogels, characterized by relatively smaller pore sizes and restricted distribution, exhibit lower equilibrium swelling degrees, suggesting a higher crosslinking efficiency of the methacrylated chitosan, resulting in a restriction of network expansion [53]. Furthermore, hydrogels with higher gelatin content (CGA_1_ and CGM_1_) show both increased pore size and equilibrium swelling, confirming that gelatin is a promoter of porosity. In contrast, formulations with increased chitosan content (CGA_3_ and CGM_3_) exhibit reduced pore size and lower equilibrium swelling, attributed to compact polymeric networks.

Overall, the results confirm that swelling behaviour is strongly dependent on pH, gelatin-to-chitosan ratio, and the type of chemical modification, with higher gelatin content and neutral pH conditions promoting increased water uptake.

### 2.4. Mechanical Properties

**Figure 4:**
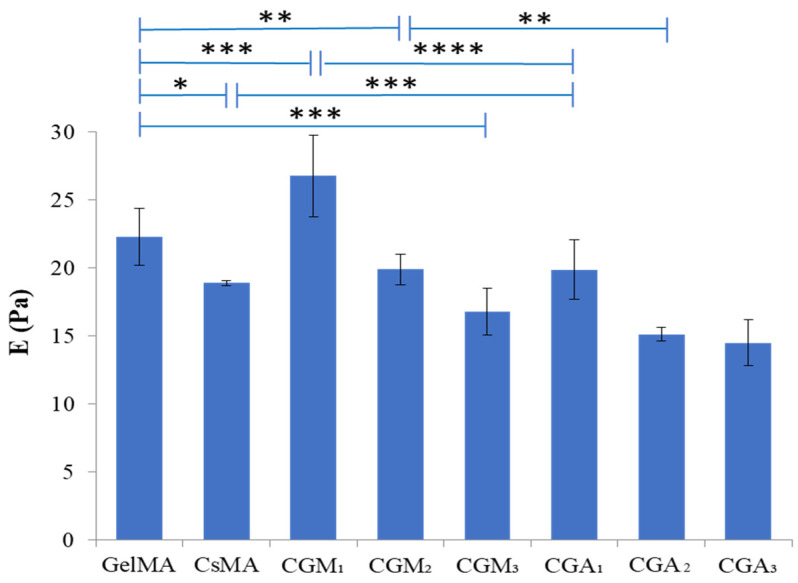
Compressive modulus (E, Pa) of the CGM and CGA hydrogels. Values were expressed as the mean ± SD (*n* = 3). * *p* < 0.05, ** *p* < 0.01, *** *p* < 0.001, **** *p* < 0.0001 (ANOVA, two-ways).

The data presented in Figure 4 indicates that the nature of the gelatin modification influences the hydrogels’ elasticity, most probably by creating shorter or larger distances between junction zones and by increasing the number of crosslinking points (in the case of methacrylate gelatin-based hydrogels). GelMA contributes to the network rigidity and, combined with methacrylated chitosan, produces a more stable and less mobile 3D network.

The compressive modulus of the hydrogels reached a value of 26.8 ± 3.1 Pa for CGM_1_. The increase in the compressive modulus could have resulted from covalent bonding and the electrostatic and hydrogen bonding interactions between methacrylate chitosan and methacrylate gelatin. In allylic gelatin-based hydrogels, the compressive modulus decreased at higher concentration, suggesting a more flexible network, able to adapt to mechanical stimulation. Moreover, these findings indicate that the mechanical properties could be adjusted by simply controlling the content of GelAly.

### 2.5. In Vitro Degradation

**Figure 5:**
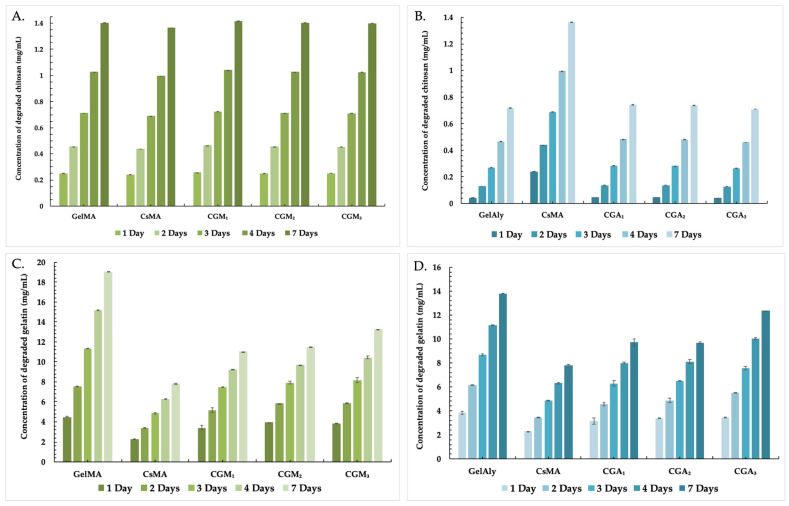
Time-dependent degradation behaviour of hydrogels determined using: the potassium ferricyanide assay (**A**,**B**), or the ninhydrin assay (**C**,**D**).

Performing the biodegradation assay of hydrogels is essential, as they function as substrates, providing three-dimensional structures with mechanical stability for cells, thus facilitating neo-tissue development. The degradation profiles of all polymeric hydrogels (Figure 5) were evaluated by direct exposure to an enzymatic mixture of collagenase and lysozyme (PBS, pH 7.2, at 37 °C, simulating the physiological conditions) and spectrophotometric measurements of the degradation products.

CsMA hydrogels exhibit a high susceptibility to structural degradation when exposed to lysozyme, as expected. On the other hand, in combination with gelatin, hydrogels exhibit a reduced breakdown rate and have the ability to preserve their integrity due to the crosslinkings between the modified groups present on both polymeric chains.

In the presence of lysozyme (Figure 5A,B), CGM and CGA samples showed reduced chitosan degradation compared to the CsMA reference, suggesting that the incorporation of gelatin and its chemical modification contributes to enhanced network stability. The degradation profiles for the components sensitive to collagenase (Figure 5C,D) revealed a significantly higher concentration of degraded gelatin compared to chitosan, for all samples. Hydrogels with an increased content of gelatin (CGA_1_, CGA_2_, CGM_1_, and CGM_2_) exhibit accelerated gelatin degradation, reflecting the higher availability of peptide bonds to hydrolytic cleavage [54].

Comparing the two spectrophotometric detection methods, gelatin degradation is more pronounced than chitosan degradation across all hydrogels, highlighting the increased sensitivity of gelatin-based components. Generally, CGM hydrogels exhibited slower degradation rates than CGA samples, which can be attributed to the crosslinking efficiency, leading to improved resistance against degradation [55,56].

Overall, these results demonstrate that the degradation behaviour of the hydrogels is strongly influenced by polymer composition, chemical functionalization, and network architecture. Higher gelatin content promotes faster gelatin degradation, while methacrylated chitosan contributes to enhanced structural stability and reduced degradation rates.

### 2.6. Drug Release Studies

The infection of skin wounds is a complex process involving the interaction of various biological pathways. Microbial contamination of a wound is the initial stage in the onset of incisional skin wound infection, which may be attributed to either endogenous or exogenous microbiota [57]. The endogenous microbiota include Escherichia coli, Staphylococcus aureus, bacilli, and Enterococcus, among others [58,59]. This form of contamination is contingent upon the surgical technique conducted. The exogenic flora primarily consists of streptococci and staphylococci, which typically originate from the operating room, including surgical tools, air, and personnel [60]. In order to avoid wound infection, the clinical use of bacitracin was approved by the United States FDA in 1948, as a curative and preventive solution for acute and chronic skin infections. Bacitracin is a group of closely related cyclic polypeptide antibiotics that can inhibit bacterial growth (bacteriostatic effect), as well as kill bacteria (bactericidal properties). Gram-positive bacteria—such as *Staphylococcus* spp., *Corynebacterium* spp., *Streptococcus* spp., *Actinomyces* spp. and *Clostridium* spp.—are generally susceptible to bacitracin [61]. The drug exhibits activity against a few Gram-negative bacteria, including *Neisseria* spp., whereas many other Gram-negative organisms demonstrate resistance. Topical bacitracin exerts its effect by inhibiting mucopeptide transport into bacterial cell walls, and can protect injured or regenerating skin [62].

**Figure 6:**
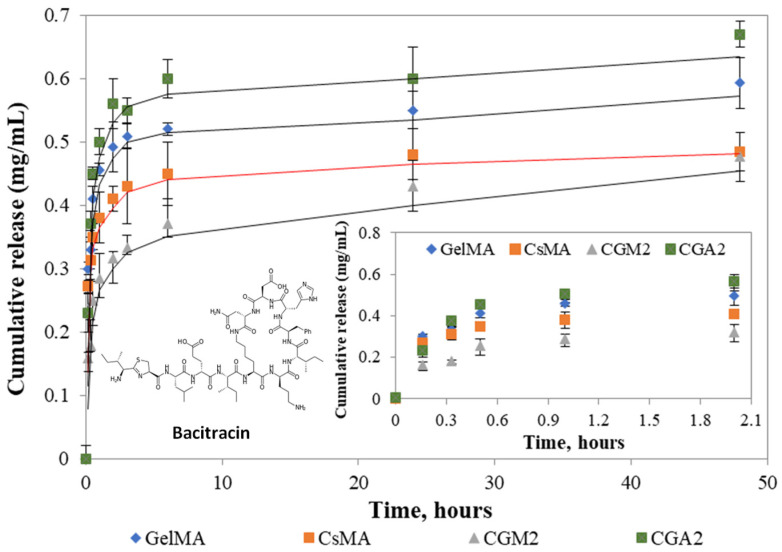
Bacitracin release profiles from hydrogels.

Bacitracin release studies were performed under physiological conditions, 37 °C and pH = 7.4, mimicking the extracellular environment. The release kinetic profiles for bacitracin are presented in Figure 6. An initial burst release of Bacitracin from all hydrogels was detected during the first 6 h, followed by a controlled, slower release. This behavior can be attributed to the three-dimensional architecture and high water content of hydrogels, which regulate drug diffusion.

The CGA2 hydrogel demonstrated the greatest release of Bacitracin; however, methacrylation-induced modification of the chitosan structure decreased the release capacity, likely as a result of reduced porosity in the hydrogel network. Bacitracin itself may actively contribute to the formation of new hydrogen bonds within the 3D network, through interactions between its -COOH and -NH_2_ groups and the hydrophilic groups present in chitosan and gelatin, leading to the formation of physical associations. Moreover, Bacitracin is a large molecule and is more difficult to release in materials with smaller pores. The data are consistent with SEM and swelling degree analyses and illustrate how the length of gelatin functional groups affects the drug release capacity of hydrogels.

It is widely recognized that drug release from hydrogels is controlled by multiple mechanisms, depending on the type of polymer, network stability, structural parameters, morphology, hydrophilicity, the characteristics of the drug, its interactions with the polymeric network, and the properties of the release medium, including diffusion, erosion, and network relaxation [63]. To investigate the release kinetics and underlying mechanism, the experimental data were analyzed using the Korsmeyer–Peppas model, as expressed in Equation (1) [64]:

(1) $$\frac{M_{t}}{M_{\infty }}=k\times t^{n},$$

where M_t_ = total amount of drug released at time “t”; $M_{\infty }$ = total amount of drug to be released; k = kinetics constant; n = diffusion exponent; and t = time (h). 

The values of diffusion coefficient *n* correspond to a release mechanism of the drug by the non-Fickian diffusion model (0.5 < *n* < 0.89) [65]. Non-Fickian diffusion, also termed abnormal diffusion, describes a time-dependent release mechanism governed by the interplay between contraction-driven release and Bacitracin diffusion rates (Table 2).

A behavior closer to Fickian diffusion was observed in the hydrogel CGA_2_. Bacitracin (BA) is a polypeptide antibiotic whose large molecular size and functional groups influence its diffusion from hydrogels, partially overlapping with the effects of network crosslinking. Hydrogel wound dressings with tailored crosslinking have been demonstrated to affect both drug release profiles, suggesting that adjustments in network density and intermolecular interactions can enable precise control over release kinetics [66].

### 2.7. Bioadhesion Tests

In tissue engineering applications, cell adhesion to polymeric scaffolds is an essential property for tissue repair and regeneration. Chitosan is a cationic biopolymer that is commonly utilized in the manufacture of bioadhesive delivery systems. Mucoadhesion is influenced by the interactions between the positive charge of amino groups of chitosan and the negative charge of the glycoproteins present on the mucosal surface; that is, the exposed groups from the polymers that compose the hydrogels will be directly implicated. In order, the -OH group, -COOH, -NH_2_, and -CH_3_ will form the strongest bonds with the cells, and this is especially observed for the hydrophilic groups due to hydrogen bonding [67].

For evaluating the bioadhesive properties of the hydrogels, texture analyzers are widely used. These instruments measure the value of detachment force (maximum force reported) and work of adhesion (effort required to separate the sample from the membrane), indicating the adhesive potential of the tested materials.

**Figure 7:**
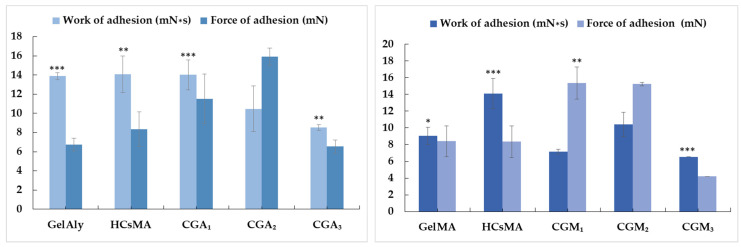
Bioadhesion properties of CGM- or CGA-based hydrogels determined as the detachment force and the work of adhesion. Values are expressed as the mean ± SD (*n* = 3). * *p* < 0.05, ** *p* < 0.01, *** *p* < 0.001 (one way ANOVA).

In vitro bioadhesion studies performed on the obtained hydrogels are represented in Figure 7. It can be observed that, when comparing the modified polymers, adhesion values for GelAly are higher than for GelMA. This effect may be attributed to the greater flexibility of the GelAly polymer compared to the more rigid GelMA. As the proportion of modified gelatin in the hydrogel increases, the adhesiveness of the scaffolds tends to decrease. This is likely due to stronger and more rigid interactions forming between the hydrogel components, specifically between the modified chitosan and the modified gelatin [68].

Being a gelatin derivative, GelMA or GelAly will maintain its cell-interactive properties, which are mostly due to the COOH, -OH groups, but also to RGD sequences, which in the presence of cells will interact with the integrin receptors, an interaction that is influenced by the mechanical properties of the scaffold [68].

### 2.8. In Vitro Cytocompatibility Results

Cytocompatibility is a significant requirement for assessing cellular responses to the hydrogels’ composition and structure, determined by the survival, metabolic activity, spreading, and proliferation of the cells. Despite the established ability of gelatin-based scaffolds to facilitate proper interaction and cell proliferation due to their peptide sequences, the structure of the polymeric network, resulting from either cross-linking or the concentration of solid polymers, may hinder cell–cell communication and functionality, ultimately leading to cell death [69]. Accordingly, the studied hydrogels were designed from biocompatible polymers, modified and crosslinked to support a cell-friendly environment.

Cell response to the presence of the hydrogels was investigated using two cell lines of fibroblasts, these cells presenting a major role in the wound healing process. Mouse fibroblasts (BALB/3T3 cell line) and primary fibroblasts were exposed for 72 h to the scaffolds, with no pH modifications observed in the culture medium in which they were immersed. GelMA, like gelatin and collagen, retains essential cell-binding motifs such as the RGD (arginine–glycine–aspartic acid) peptide, a crucial characteristic that can enhance cell viability and promote cellular activity, particularly cell proliferation, migration, and differentiation.

It was reported by Lloyd et al. [70] that chitosan can reduce scar formation during the wound healing process by reducing fibrin production and enhancing blood coagulation through a unique mechanism involving the interaction between erythrocyte cell membranes and chitosan filaments.

**Figure 8:**
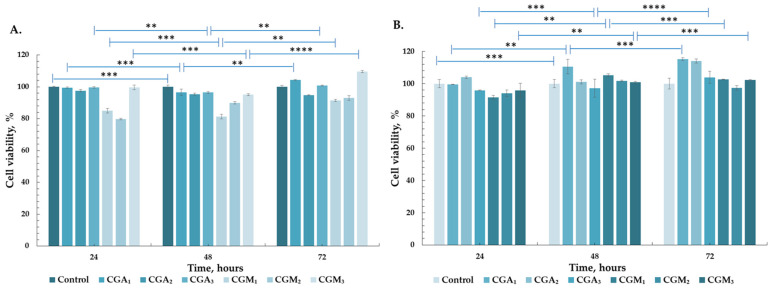
Cell viability data from the MTT assays for CsMA_GelMA/GelAly based hydrogels, tests on primary fibroblasts (**A**) and BALB/3T3 cell line (**B**). ** *p* < 0.01, *** *p* < 0.001, **** *p* < 0.0001; *n* = 3.

Figure 8A demonstrates that CGM and CGA hydrogels maintained cell viability over 80% in MTT experiments, in the first 24 h, reaching over 90% and even 100% cell viability after 72 h of direct cell–hydrogel contact in the experiments using primary fibroblasts. The viability values for the BALB/3T3 cell line (Figure 8B) in CGA scaffolds, assessed 24 h post-contact, was higher than 95% for CGA_1_ and CGA_3_, 100% for CGA_2_, and 90% for all CGM scaffolds, with a significant increase observed in all groups after 72 h (values ranging from 97.36% to 115.26% after three days of culture). The study of Klak et al. showed that methacrylated chitosan presents similar high biocompatibility when compared to non-modified chitosan [71], while hydrogels based on methacrylated gelatin presented cell viabilities over 90% when tested in the presence of fibroblasts [72]. Our results indicate the same high cell viability when testing the modified polymer-based hydrogels. Consequently, the cell viability assay demonstrated that the CGM and CGA scaffolds are appropriate for tissue engineering applications, the obtained values fulfilling the requirements of ISO 10993–5 standard [73].

**Figure 9:**
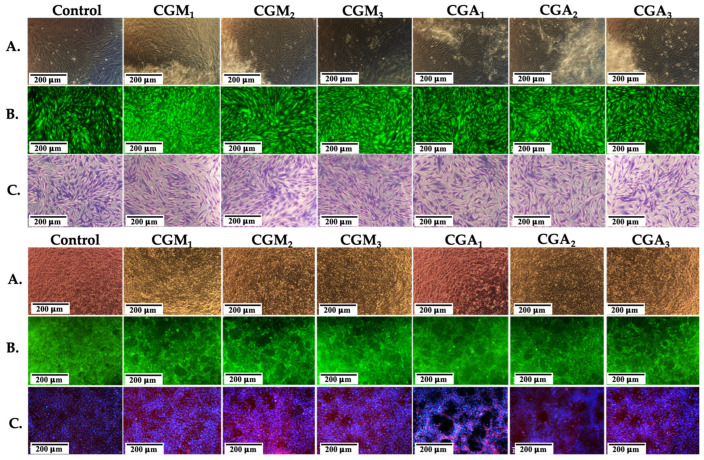
Viable cells and fixed cells, respectively, after 72 h of cell culturing with CGM and CGA hydrogels for primary (**A**—light microscopy, **B**—Calcein-AM, **C**—Giemsa/May–Grunwald staining) and BALB/3T3 fibroblasts (**A**—light microscopy, **B**—Calcein-AM, **C**—DAPI-Rhodamine/Phalloidin staining).

The tested fibroblasts present normal, specific elongated morphology after 72 h contact with the scaffolds, with similar density as for the control, their proliferation capacity being maintained until achieving a nearly confluent monolayer (Figure 9).

Elongation, intercellular interaction, and cell division were observed under the tested culture conditions, suggesting that CGM and CGA scaffolds will not hinder normal cellular signaling. Figure 8 presents fibroblast morphology evaluated using the fluorescent marker Calcein AM, May–Grunwald/Giemsa staining, and DAPI- Rhodamine Phalloidin staining.

Similar morphologies can be observed when compared to the control and normal extended cytoskeleton of the cells. Gelatin possesses well-known cell-friendly features, biocompatibility, and bioactive patterns preserved even after the modification reactions with allyl glycidyl ether (GelAly) [31] or through methacrylation (GelMA){Citation}. The incorporation of methacryloyl or allylic side groups enables the GelMA/GelAly molecule to crosslink with CsMA, resulting in the formation of stable 3D structures at physiological temperature.

### 2.9. Wound Healing Assay

Wound healing is a complex process comprising four main phases, including hemostasis, inflammation, cell proliferation, and tissue remodeling, facilitated by cellular signaling in conjunction with important interactions between the cells and the structural polymers of the extracellular matrix (ECM) [74]. Cellular signaling is promoted by growth factors, interleukins, chemokines, and cytokines, assuring an efficient interplay that will facilitate tissue restoration. An efficient communication will be assured by proper scaffold properties, in particular the presence of peptide sequences specific for collagen-derived compounds, such as gelatin. Chitosan and its derivatives are suitable candidates for wound healing applications due to their implication in promoting fibroblast proliferation and migration through the PI3K/AKT signaling pathway, biocompatibility, antimicrobial, and anti-inflammatory properties [75,76,77,78]. Gelatin derivatives enhance the beneficial properties of chitosan in wound healing applications as they promote cell adhesion, migration, and support extracellular matrix formation. Other studies, in which thiolated quaternary ammonium chitosan derivatives were tested, determined an increase in fibroblast migration when compared with untreated controls, while chitosan-gelatin nanofiber scaffolds determined in vitro wound closure in 48 h [79,80].

**Figure 10:**
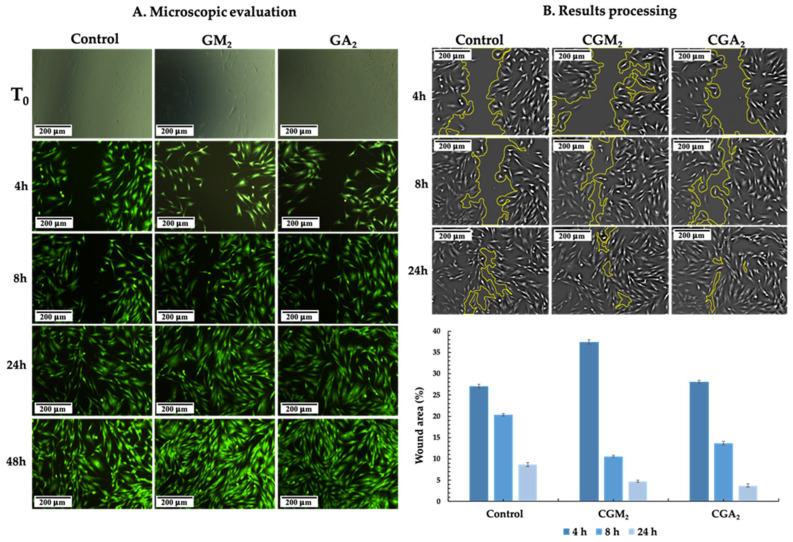
(**A**) In vitro wound closure at different time intervals for the cells exposed to CGM_2_ and CGA_2_ hydrogels, compared to the control. (**B**) Wound area calculated for the 4 h, 8 h, and 24 h time intervals.

Various methods have been established to assess a material’s capacity to regenerate biological tissues, ranging from in vitro wound scratch assays to in vivo investigations. In this study, the scratch test was performed in order to examine the hydrogels’ effects on cells’ ability to migrate and cover the artificially formed gap. The in vitro wound healing effect of CsMA_GelMA or GelAly polymeric hydrogels was observed in a time-dependent (0–48 h) manner following the migration and proliferation of the normal human dermal fibroblasts cell line. The impact of the materials on wound healing was evaluated by microscopic examination of the wounded areas, employing Calcein-AM staining at distinct time intervals (Figure 10).

For this assay, two types of materials were chosen, CGM_2_ and CGA_2_, with an equilibrated ratio between the polymers in the compositions. It can be observed from Figure 10 that there are differences in the migration behaviour between the cells exposed to the hydrogels and the control, especially after the first 8 h, a time period that reflects the migration of the cells more accurately, without cell division. After 8 h, gap area reduction was with 26.8% for CGM_2_-exposed cells and 14.4% for CGA_2_-exposed cells, compared to 6.7% for the control. Gradual migration of the cells was observed until 24 h, while at 48 h, there was a complete closure of the gap for the cells exposed to both types of tested hydrogels. These results show that the synergistic interaction between the polymeric components of the hydrogels can enhance cell migration, facilitating expedited wound closure.

### 2.10. Anti-Inflammatory and Antioxidant Properties

**Figure 11:**
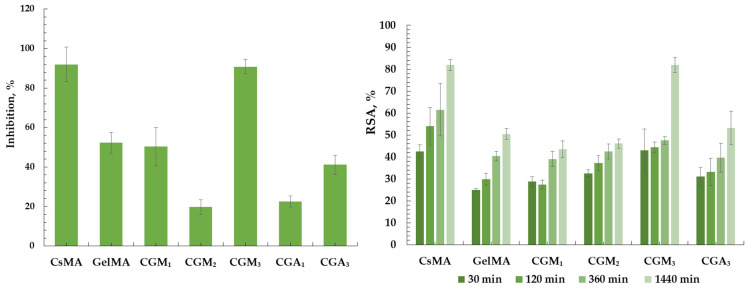
The percentage of protein denaturation inhibition (Inhibition, %) and antioxidant properties (RSA, %) of CGM or CGA-based hydrogels.

Figure 11 illustrates the hydrogels’ biological performances, evaluated through a combination of anti-inflammatory and antioxidant properties. The results clearly indicate that the hydrogels’ behaviour in a biological environment is influenced by the ratio between methacrylated chitosan (CsMA) and modified gelatin derivatives (GelMA or GA), with consistent trends observed in both anti-inflammatory and antioxidant assays. Chitosan-rich formulations (CsMA and CGM_3_) showed the highest inhibition of protein denaturation (~90%), confirming the key role of chitosan in protein stabilization and its well-known anti-inflammatory potential [81,82]. In contrast, systems based predominantly on methacrylated gelatin (GelMA and CGM_1_) exhibited only moderate inhibition (~50%), while the GA-based formulation with lower chitosan content (CGA_1_) showed reduced activity (~22%), suggesting that some combination of chitosan and gelatin, regardless of methacrylation or allylation, contributes mainly to structural properties [83,84].

Allyl-functionalized gelatin (GA) exerts a noticeable influence on CGA hydrogel formulations. The presence of allyl groups influences the network architecture, crosslinking behaviour, and diffusion processes [85,86]. As a result, increasing the chitosan content (CGA_3_ vs. CGA_1_) improves both anti-inflammatory and antioxidant performance, although the effect is less pronounced than in CGM systems, due to the enhanced polymer–polymer interactions and microenvironment effects.

A similar trend was observed in the antioxidant assay, where all samples showed a time-dependent increase in radical scavenging activity, reaching maximum values after 24 h, consistent with diffusion-controlled mechanisms. Also, chitosan-rich hydrogels exhibited superior activity, which can be attributed to the presence of amino and hydroxyl groups capable of donating electrons or hydrogen atoms to neutralize free radicals, while gelatin-based systems remained less effective. CGM_2_ exhibited intermediate behaviour, likely reflecting a balance between structural integrity and functional group availability. The limited differences between CGA_1_ and CGA_3_ suggest that, beyond composition, network architecture and diffusion constraints may influence antioxidant efficiency [87,88].

Overall, the strong correlation between antioxidant capacity and protein stabilization suggests that the same functional groups of chitosan (–NH_2_ and –OH) are responsible for both radical scavenging and anti-inflammatory effects [81,89].

## 3. Conclusions

In the present study, methacrylated chitosan was combined in different formulations with methacrylated gelatin and allylic gelatin, and photocrosslinked to prepare a hydrogel with potential applications as a wound dressing. FT-IR analysis, the swelling, and the mechanical properties confirmed the 3D networking and the compatibility between the hydrogel components. Combined with degradation tests and SEM analyses, the results indicate that the allylic gelatin-based hydrogels present larger pores and a stronger pH-responsive swelling behaviour compared to methacrylated gelatin-based samples, reflecting the higher flexibility of allylic gelatin networks. The hydrogels release bacitracin during the first six hours, with a release profile that follows a non-Fickian diffusion mechanism. Bioadhesion improved properties were observed for more flexible allylic gelatin—based materials, increasing the potential in wound healing applications of these hydrogels. The cytocompatibility of the hydrogels was tested using two different lines of fibroblasts, an important type of cell implicated in the process of wound healing. The polymer modifications do not compromise cytocompatibility, but offer a different possibility of crosslinking, the results showing high cell viability after 72 h of direct contact with the hydrogels. In vitro simulating wound healing tests demonstrated that prepared hydrogels facilitate cell migration compared to the control and determined complete wound closure. All formulated hydrogels exhibit antioxidant capacity and protein stabilization properties, which are attributed to the presence of chitosan in their composition. The studied hydrogels incorporate polymers that maintain the cell-friendly characteristics of chitosan and gelatin, while targeted chemical modifications facilitate the development of new network structures with potential use in wound healing and drug delivery.

## 4. Materials and Methods

### 4.1. Materials

Gelatin (Gelatin A, from porcine skin, gel strength ~300 g Bloom) and high molecular weight chitosan (Mw = 31–37.5 × 10^4^ Da) were purchased from Sigma-Aldrich, Burlington, MA, USA. Methacrylated gelatin (degree of substitution: 74.78% (^1^HNMR), 66.52% (TNBS method), 68.07 ± 0.01% (ninhydrin method) previously demonstrated [67] and methacrylated chitosan (CsMA, degree of substitution about 42.2% (^1^HNMR), 47.50 ± 1.83% (TNBS method), and 52.94 ± 4.50% (ninhydrin method) were prepared using the method described in the literature [90]. The degree of modification determined from the ^1^HNMR spectra is comparable to values reported in the literature for GelMA and CsMA synthesized using methacrylic anhydride [91,92].

In order to analyze the behaviour of the polymeric scaffolds in physiological conditions, different types of reagents were used: lysozyme from chicken egg (105.000 U/mg), collagenase (type I, activity ≥ 125 units/mg, US Biological), ninhydrin, and potassium ferricyanide solution. Moreover, different phosphate-buffered saline (PBS) solutions, 0.01 M, pH = 7.2 and 5.4, prepared from monosodium phosphate (NaH_2_PO_4_·2H_2_O) and disodium phosphate (Na_2_HPO_4_), both from Sigma-Aldrich, Hamburg, Germany, were used for the preparation of gels and various characterization assays.

The cytotoxicity tests and wound healing assay were performed using human primary fibroblasts (NHDF, cell line, Gibco™ Thermo Fisher Scientific, Waltham, MA, USA) and mouse BALB/3T3 fibroblasts (ATCC, Rockville, MD, USA) and specific reagents for cell cultures: Hank’s Balanced Salt Solution (HBSS), Dulbecco′s Modified Eagle′s Medium/Nutrient Mixture F-12 Ham (DMEM) and DMEM High Glucose, FBS (fetal bovine serum, sterile-filtered, suitable for cell culture), P/S/N (penicillin/streptomycin/neomycin solution) and MTT (3-(4.5-dimethyl-2-thiazolyl)-2.5-diphenyl-2H-tetrazolium bromide), Calcein AM, DAPI and Rhodamine Phalloidine, from Sigma-Aldrich, Hamburg, Germany.

All other solvents and chemicals were obtained from Sigma-Aldrich, Hamburg, Germany, and used as received.

### 4.2. Hydrogels Preparation

In order to obtain crosslinked hydrogels, the polymeric solutions (3%, wt/wt) were prepared by dissolving chitosan/gelatin and their derivatives in phosphate-buffered solution (PBS, 0.01 M, pH 7.2), mixed at different ratios (as presented in Table 3) and homogenized by stirring (200 rpm, 60 min, magnetic stirrer).

**Figure 12:**
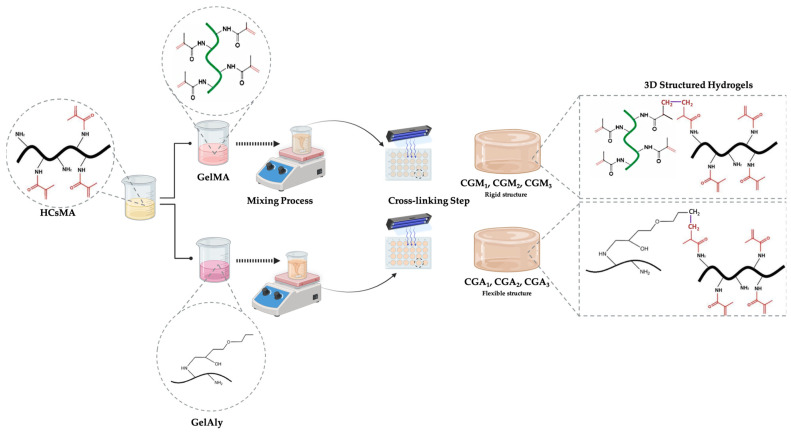
Schematic diagram of CsMA/GelMA and CsMA/GelAly hydrogels preparation.

Subsequently, 500 µL of Irgacure 2959 photoinitiator (0.5%, prepared in 0.01 M PBS at pH 7.2) was added to each polymer formulation. Irgacure 2959 is a cytocompatible UV photoinitiator, which was included to induce the rearrangement of molecules and produce free radical intermediates by visible light in order to create co-networking hydrogels. As illustrated in Figure 12, hydrogel samples with a diameter of 15 mm were obtained by casting them in the wells of culture plates. The photocrosslinking process was carried out by exposing the samples to 365 nm UV light using a Herolab lamp (15 W tube; Herolab, Wiesloch, Germany) for 10 min, with the light source positioned approximately 10 cm from the samples. Then, the obtained 3D structures were freeze-dried (freeze-drier from Labconco (Kansas City, MO, USA), at a rate of 1 °C/min until the temperature reached −54 °C and a vacuum of 41 mTorr) for further characterization.

### 4.3. Hydrogels Characterization

#### 4.3.1. FT-IR Spectroscopy

FT-IR spectra were recorded using a Vertex Bruker Spectrometer (Wien, Austria) in an absorption mode range (400 to 4000 cm^−1^). Each sample was ground using potassium bromide (KBr) powder and compressed into a disc form, which was analyzed to generate spectra at a resolution of 4 cm^−1^, averaging 64 scans. The results were plotted as graphs using OriginLab software 8.5.

#### 4.3.2. Scanning Electron Microscopy

An evaluation of the hydrogels’ porosity was performed through scanning electron microscopy (SEM) analysis. Cross-sections of the hydrogels were analyzed using a Hitachi SU-1510 scanning electron microscope (Hitachi SU-1510, Hitachi Company, Tokyo, Japan). Cross-sections of dried materials were mounted and fixed on an aluminum stub, then coated with a 7 nm gold layer (Cressington 108 Sputter Coater, High Wycombe, UK). The resulting images were subsequently analyzed using ImageJ software, version 1.54g.

#### 4.3.3. In Vitro Absorption of Simulated Body Fluids

The tests were performed using a volumetric method, under the following experimental conditions: the samples were immersed in phosphate buffer solutions (PBS of pH 7.2 or pH 5.4, 0.01 M), incubated at 37 °C, using QIAquick RSpin Columns 50 (Qiagen, Hilden, Germany), Ø = 10 mm, and connected to a micro syringe (1ml, Qiagen, Hilden, Germany). During the analysis, the volume of phosphate-buffered saline solution retained in the hydrogel structure was calculated using the following equation:

(2) $$SD=\frac{W_{t}-W_{0}}{W_{0}}\times 100\left(\%\right),$$

where *W*_0_ is the initial weight of the scaffold and *W_t_* is the weight (g) of the swollen sample, calculated as *W*_0_ + *W_abs_* (*W_abs_* represents the amount of retained PBS; PBS density ~1 g/mL). The experiments were performed in triplicate, and the results are expressed as mean ± standard deviation.

#### 4.3.4. Mechanical Features

Hydrogels’ elasticity moduli were calculated from compression tests performed on a TA-XT2 Plus Texture analyzer (Stable Microsystems, Godalming, UK). The cylinder had a diameter of 12 mm, and the compression speed was 1 mm/s. We evaluated three replicates of each hydrogel at a pre-test speed of 1 mm/s, a compression speed of 2 mm/s, and a deformation of 0.25%. The slopes of stress–strain curves at small deformations were used to calculate the apparent compression modulus.

#### 4.3.5. In Vitro Degradation Studies

Enzymatic degradation studies were performed to evaluate the stability and biodegradability of the hydrogel networks under physiologically relevant conditions. This assessment provides essential information regarding polymer breakdown kinetics and the suitability of the hydrogels for biomedical applications requiring controlled degradation [93].

Each material (20 mg) was immersed in 3 mL PBS (pH = 7.2, 0.01 M), containing an enzymatic mixture (1200 µg/mL lysozyme and 0.01% collagenase), in a dialysis membrane (MWCO = 14,000 Da). Further, the membrane was immersed in 10 mL PBS (pH = 7.2, 0.01 M) and incubated at 37 °C. In order to determine the time-dependent degradation due to the enzymatic susceptibility of the scaffolds, colorimetric spectrophotometric measurements were employed. In total, 1 mL of solution was sampled at different time intervals. The concentration of saccharide reduced units produced after chitosan degradation was measured using the potassium ferricyanide method, which involved mixing the extracted solution with 0.05% potassium ferricyanide solution in 0.5 M Na_2_CO_3_ solution, boiling it for 15 min, and then diluting it with Na_2_CO_3_. The degraded gelatin was measured using the ninhydrin method. This assay involved mixing the extracted sample with 1 mL of ninhydrin solution, boiling for 20 min, and dilution with a distilled water solution/2-propanol (1:1). The absorbance was measured at 420 nm for reduced saccharide concentration and 570 nm for amino acid concentration, using a PharmaSpecUV-1700 spectrophotometer (Shimadzu, Duisburg, Germany).

#### 4.3.6. Drug Loading and Drug Release Studies

The dried hydrogel samples were immersed in an aqueous solution of Bacitracin (10 mg/mL, distilled water) and kept for 24 h at room temperature in the dark. The loaded samples were freeze-dried for 24 h (−54 °C and a vacuum value of 41 mTorr, Labconco (USA) freeze-drier). The drug release experiment was carried out by immersing the drug-incorporated hydrogels into 1 mL solution of PBS (pH = 7.2, 0.01 M), followed by incubation at 37 °C. Periodically, 2 μL of the release medium was withdrawn, and the amount of Bacitracin was measured spectrophotometrically at a wavelength of 254 nm using a DeNovix DS-11 NanoDrop (Thermo Fisher Scientific, Waltham, MA, USA). The cumulative release data were obtained using a calibration curve for Bacitracin.

#### 4.3.7. Bioadhesion Test

A TA.XT plus^®^ analyzer from Stable Micro Systems (Godalming, UK) was used to evaluate adhesive properties in the form of adhesion force and total work of adhesion. The bioadhesion experiments employed a cellulose membrane as a simulated biological interface, prepared prior to testing by boiling and cooling, as described in earlier protocols [94]. Testing bioadhesion on cellulose membrane has shown good correlations when compared to animal mucosa [95]. The tests were performed using a phosphate-buffered solution with a pH of 7.2, 0.01 M (100 µL PBS added to each sample), which was added onto the fixed membrane, the system operating at 37 °C. The hydrogel samples, with a diameter of 8 mm, were brought into contact with the membrane at a controlled speed of 1 mm/s, maintained for 30 s, then detached until separation, with four determinations for each sample. The data, in the form of maximum detachment force and work of adhesion, were analyzed using the specific Texture Exponent software, version 32.

#### 4.3.8. In Vitro Cytocompatibility Studies

##### MTT Assay and Cell Morphology Evaluation

For analyzing hydrogels biocompatibility human primary fibroblasts (NHDF cell line) and mouse fibroblasts (BALB/3T3 cell line) were incubated for 24 h in specific conditions: 5% CO_2_, 37 °C, 95% relative humidity, in Dulbecco′s Modified Eagle′s Medium/Nutrient Mixture F-12 Ham, with L-glutamine supplemented with 10% FBS and 1% P/S/N, in 48 wells plates, 1 × 10^4^ cells/well.

Cell viability was assessed using a direct contact MTT assay, according to ISO 10993-5:2009 [73]. The hydrogels (CGM_1_, CGM_2_, CGM_3,_ CGA_1_, CGA_2_, CGA_3_) were cut with a biopsy punch, then sterilized by exposure to UV light (λ = 262 nm) for 30 min on each side, followed by 24 h equilibration in culture medium, in 24-well plates. The samples were then added to the prepared 48-well plates, in triplicate, for both cell lines. Wells in which the cells were not exposed to hydrogels were used as a reference for the quantification of the cellular viability. After 24, 48, and 72 h, the hydrogel samples were removed, and the media was replaced with MTT solution (5% concentration in simple DMEM) and were incubated at 37 °C for 3 h. Formed formazan crystals were dissolved using DMSO, 500 µL/well. The absorbance of the resulting solution (purple color) was measured at a wavelength of 570 nm using a Tecan Sunrise^TM^ Plate Reader (Tecan Trading AG, Männedorf, Switzerland). The absorbances from the experimental wells were compared to the control wells that did not include any pieces of material. The cell viability was represented by the estimated ratio:

(3) $$V\;=\frac{A_{s}}{A_{c}}\times 100\;(\%).$$

Here, V—cell’s viability; *A_s_*—sample absorbance; and *A_c_*—control absorbance. Each result represents the mean viability and standard deviation.

The experiments were performed in triplicate (*n* = 3), and the results were expressed as ± standard deviation (*SD*). Data were analyzed using two-way ANOVA and Tukey’s post hoc analysis in order to detect the variables’ significance, and *p* < 0.01 was considered significant.

In addition to the MTT test, different staining, specific for live or fixed cells, was applied onto the hydrogel-exposed cells in order to evaluate the influence of the scaffolds on cell morphology. Calcein-AM staining [96] involved a fluorescent cell-permeable derivative of calcein. The modification of the carboxylic acid groups on calcein with acetoxymethyl (AM) ester groups increases the molecule’s hydrophobicity and facilitates cell membrane penetration and the evaluation of the cells’ metabolic state [97]. For this, a Calcein-AM solution with a concentration of 2 µM was prepared in HBBS (with Ca^2+^, Mg^2+^). This was added to the tested wells and incubated at 37 °C for 30 min.

DAPI-Rhodamine Phallodin was used to evaluate the nucleus integrity and cytoskeleton components as a result of the interaction with the hydrogels. DAPI is a blue DNA-binding dye that increases its quantum yield when bound to double-stranded DNA [98]. Rhodamine serves as a fluorescent marker in the orange-red spectrum, allowing the observation of the filamentous network within the cell, represented by actin filaments [99]. For this, cells were fixed with formic aldehyde solution for 30 min, permeabilized with Triton X-100, and rinsed with PBS solution (three times). Rhodamine–Phalloidin dye was applied and left for 2 h, followed by DAPI solution with an incubation time of 30 min at 25 °C. The cells were then rinsed twice with PBS and examined.

For the May–Grunwald/Giemsa method, the staining was applied to fixed cells, first May–Grunwald for 3 min, then alkaline Giemsa for 25 min, after which the cells were washed with double-distilled water and analyzed.

All the images were acquired using a Leica DM IL LED Inverted Microscope (Leica Microsystems GmbH, Wetzlar, Germany) with a Phase Contrast System and specific fluorescence filters, using a 10× objective.

##### Wound Healing Assay

The cell migration investigation, which illustrates the hydrogels’ wound-healing properties, was assessed using a modified scratch assay protocol [100,101]. Normal human fibroblasts (NHDF cell line) were used to evaluate the in vitro regeneration properties of hydrogels. The cells were prepared under identical conditions as in cytotoxicity assays (48-well culture plate at a density of 12 × 10^3^ cells/well, for 48 h, in DMEM-HAM F12 complete media, at 37 °C in a 5% CO_2_ environment to facilitate cell adhesion to the well surface). At 80% cell confluence, a gap in the cells’ layer was methodically scraped with a sterile 200 µL pipette tip to generate uniform “wounds”. The remaining adherent cells were rinsed twice with HBSS (lacking Ca^2+^ and Mg^2+^) to eliminate the detached cells. DMEM-HAM F12 was added to each well, followed by the acquisition of microscopic images to establish the time zero (T_0_) of the experiment. Sterilized and pre-equilibrated hydrogels (CGM_2_ and CGA_2_) were applied in the wells with the scratched cellular layers.

The cell migration process was monitored at different time intervals (4 h, 8 h, 24 h, and 48 h) by staining cells using Calcein AM reagent, following the protocol previously described, and images were recorded using a Leica DM IL LED Inverted Microscope with a Phase Contrast System and Fluorescence at a 10× objective. The wound area was evaluated after image acquisition and processing in ImageJ Software (2020, MRI wound healing tool).

#### 4.3.9. Biological Properties

##### Inhibition of Protein Denaturation

The procedure outlined by Obluchinskaya [102] was used to test protein denaturation, though alongside several changes. In short, 2 mg of material was kept at (27 ± 1) °C for 15 min in a solution of 2.8 mL of phosphate-buffered saline (pH 6.4) and 2 mL of bovine albumin (1 mM, in phosphate-buffered saline, pH 6.4), and then 10 min at 70 °C in a water bath for protein denaturation. After that, each solution was cooled, and its absorbance value was measured at 660 nm using a spectrophotometer DeNovix DS-11 NanoDrop (Thermo Fisher Scientific, Waltham, MA, USA). The percentage of denaturation inhibition was calculated using the control group that did not contain any material.

(4) $$Inhibition\left(\%\right)=\left(\frac{A_{s}-A_{c}}{A_{c}}\right)\times 100,$$

where *A_s_* is the absorbance value of the evaluated solution with the scaffold and *A_c_* is the absorbance value of the control solution (without hydrogel). Each experiment was conducted in triplicate, and the mean was calculated.

##### Antioxidant Activity

Hydrogels’ antioxidant activity was evaluated through the DPPH radical-scavenging assay, being measured according to the reference, with some modifications [103,104]. Briefly, 1 mL of hydrogel solution (2 mg/mL, PBS pH 7.2, 0.01 M) was incubated with 1 mL of 0.1 mm DPPH solution, 950 µL of buffer solution of Triss HCl (pH 8), and 1 mL ethanol. At different time intervals (30, 120, 360, and 1440 min), the absorbance of 2 µL of the mixture was spectrophotometrically measured at a wavelength of 517 nm, using a DeNovix DS-11 NanoDrop (Thermo Fisher Scientific, Waltham, MA, USA). The DPPH free radical scavenging activity (RSA%) was calculated according to Equation (5):

(5) $$RSA\%=\frac{A_{r}{-A}_{s}}{A_{r}}\times 100$$

where A_r_—the absorbance of the DPPH solution (0.1 mM) used as a control solution (based on a mixture of 1 mL DPPH solution, 1 mL distilled water (hydrogel solution substitution), 950 µL Buffer Solution of Tris HCl pH 8, and 1 mL ethanol), A_s_—the absorbance of the solutions incubated with the hydrogel samples.

##### Statistical Analysis

Statistical evaluation was performed using two-way ANOVA with Tukey’s post hoc analysis. A *p*-value < 0.05 was considered statistically significant. Results are expressed as mean ± SD based on at least three independent experiments.

## Figures and Tables

**Table 1:** Pore size variations with hydrogel composition.

CsMA		GelMA		GelAly		CGM_2_		CGA_2_	
Min	Max	Min	Max	Min	Max	Min	Max	Min	Max
35.25 ± 3.17	165.17 ± 2.03	25.51 ± 1.97	205.10 ± 5.21	28.17 ± 3.88	210.70 ± 2.36	15.57 ± 5.71	95.91 ± 7.65	45.81 ± 3.44	220.51 ± 2.94
Distribution type									
Unimodal		Unimodal		Slightly bimodal		Unimodal		Unimodal	

**Table 2:** Model constants and R-squared of Bacitracin release fitted to the Korsmeyer–Peppas model.

Parameters	GelMA	CsMA	CGM_2_	CGA_2_
k	0.4243	0.7414	0.3419	0.6601
*n*	0.7585	0.8552	0.7339	0.6763
R_2_	0.9896	0.9958	0.9882	0.9791

**Table 3:** Hydrogels composition.

Hydrogel	CsMA:GelMA	Hydrogel	CsMA:GelAly	Irgacure 2959 (%, Reported to Polymer)
GelMA	0:1	GelAly	0:1	1.5
CGM_1_	1:3	CGA_1_	1:3	
CGM_2_	1:1	CGA_2_	1:1	
CGM_3_	3:1	CGA_3_	3:1	
CsMA	1:0	CsMA	1:0	

## Data Availability

The data presented in this study are available on request from the corresponding author.
